# The Integration of Cognitive Remediation Therapy into the Whole Psychosocial Rehabilitation Process: An Evidence-Based and Person-Centered Approach

**DOI:** 10.1155/2012/386895

**Published:** 2012-07-02

**Authors:** Rafael Penadés, Rosa Catalán, Núria Pujol, Guillem Masana, Clemente García-Rizo, Miquel Bernardo

**Affiliations:** ^1^Department of Psychiatry and Clinical Psychobiology, University of Barcelona, 08007 Barcelona, Spain; ^2^Institut d'Investigacions Biomèdiques August Pi i Sunyer (IDIBAPS), 08036 Barcelona, Spain; ^3^Centro de Investigación Biomédica en Red de Salud Mental (CIBERSAM), 28007 Madrid, Spain; ^4^Institut Clínic de Neurociènces (ICN), Hospital Clínic Barcelona, 08036 Barcelona, Spain

## Abstract

Cognitive remediation therapies seem to ameliorate cognitive impairments in patients with schizophrenia. Interestingly, some improvement in daily functioning can also be expected as a result. However, to achieve these results it is necessary that cognitive remediation is carried out in the context of broader psychosocial rehabilitation involving the learning of other communication, social, and self-control skills. Unfortunately, little is known about how to integrate these different rehabilitation tools in broader rehabilitation programs. Based on both the neurocognitive behavioral approach and the action theory framework, a hierarchical flowchart is represented in this paper to integrate CRT with other evidence-based psychological therapies in outpatient settings. Finally, some evidence is provided in which cognitive abilities need to be targeted in remediation programs to improve functioning. In summary, to improve daily functioning, according to these studies, cognitive remediation needs to include the teaching of some cognitive strategies that target executive skills.

## 1. Introduction

Cognitive remediation therapy (CRT) has been defined as a behavioral training-based intervention that aims to improve cognitive processes (attention, memory, executive function, social cognition, or metacognition) with the general aim of durable benefits in community functioning (Cognitive Remediation Experts Workshop, Florence, April, 2010). Nonetheless, the majority of empirical findings on cognitive remediation therapies, including the meta-analysis, challenge the assumption that simply improving cognitive functioning in schizophrenia will spontaneously lead to better psychosocial outcomes. Moreover, the results of previous studies suggest that cognitive recovery is probably the best option to optimize the response of some patients to psychiatric rehabilitation programs. So CRT is not likely to be implemented as a stand-alone therapy but as a part of a broader psychosocial rehabilitation program. Unfortunately, little is known about how to integrate the different rehabilitation tools in a broader rehabilitation program. Regrettably, they are neither standardized nor available in routine care in the majority of clinical settings. The aim of this paper is to provide an evidence-based, person-centered method for integrating CRT into the psychosocial rehabilitation process in outpatient settings. 

## 2. Effects of Cognitive Remediation on ****Cognition and Functioning

Fortunately, convincing data about CRT efficacy in cognition and functioning can currently be found in meta-analytic studies. McGurk et al. [1] carried out a meta-analytic study showing that cognitive remediation is an effective treatment for improving cognitive impairments in schizophrenic patients, obtaining a moderate effect size in cognitive measures (Cohen's *d* = 0.51). Interestingly, cognitive remediation also seemed to produce improvement in social functioning. Although the improvement was a somewhat smaller change (Cohen's *d* = 0.36), a positive effect on symptoms was also found, suggesting that there is a reduction in symptoms after rehabilitation, although the effect size is now considered to be only small (Cohen's *d* = 0.28). As such, the study provided the first meta-analytic evidence for the impact of cognitive remediation in domains other than cognition. Furthermore, an intuitive but previously undemonstrated hypothesis was revealed. By adding cognitive remediation therapy to psychosocial rehabilitation, functional outcomes improved significantly. For instance, by adding cognitive remediation to vocational rehabilitation work, performance was improved and a higher level of work performance and longer-lasting employment was generally achieved. By and large, cognitive remediation impacts on functioning only when the intervention is part of a broader psychosocial rehabilitation program. In other words, the effects of cognitive remediation therapies are higher (Cohen's *d* = 0.47) when acting as part of broader psychosocial rehabilitation than when applying cognitive remediation therapy as an isolated intervention (Cohen's *d* = 0.05). On the other hand, CRT has been compared with active controls such as occupational therapy [2], work therapy [3], or leisure group [4], showing similar group differences to those observed when compared with nonactive controls such as support group [5], watching videos [6], or standard care. Another interesting finding is that cognitive remediation programs were more efficacious when based on strategy approach (Cohen's *d* = 0.62) than when they were based on progressive exercises or repeated practice (Cohen's *d* = 0.24). Strategy approach involves an explicit focus on teaching active cognitive strategies that target memory and executive functions. Typically, it is based on teaching methods including chunking information to facilitate recall and problem-solving skills to facilitate sequencing or planning. 

**Figure 1:**
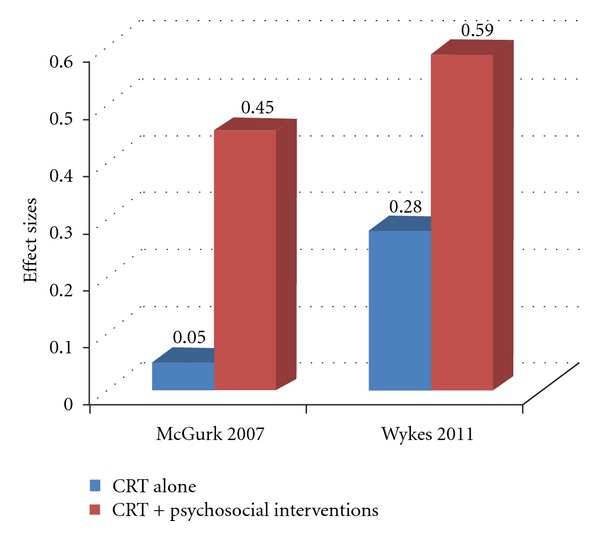
Effect sizes (Cohen's d) of cognitive remediation on functioning. Data from the meta-analytic studies by McGurk et al. [1] and Wykes et al. [2]. CRT: cognitive remediation therapy.

Recently, Wykes et al. [7] carried out another meta-analytic study with similar results. This study is based on 109 reports of 40 studies in which more than 70% of the participants had a diagnosis of schizophrenia. The meta-analysis, with 2,104 participants, confirms the durable effects of the CRT intervention on global cognition and functioning. As expected, the symptom effect was small and, unfortunately, disappeared at follow-up assessment. Surprisingly, no treatment element (remediation approach, duration, computer use, etc.) was associated with cognitive outcome. Regarding the stage of the illness, CRT seemed to be more effective when patients were clinically stable. Similarly to the former meta-analysis, significantly stronger effects on functioning were found when cognitive remediation therapy was provided together with other psychiatric rehabilitation, and a much larger effect was present when a strategic approach was adopted together with adjunctive rehabilitation (Figure 1). 

## 3. Integrating CRT with Other Psychological Therapies

Unfortunately, little is known about how to integrate CRT and the various rehabilitation tools in broader rehabilitation programs. Most relevant clinical guidelines such as PORT [8] or NICE [9] and different meta-analytic studies showed that a variety of psychosocial interventions, including cognitive behavioral psychological interventions, skills training, family interventions, and supported employment all have a convincing amount of evidence that supports their implementation in psychosocial rehabilitation programs for individuals diagnosed with schizophrenia. 

Thus, with the basic aim of providing a method for the implementation of CRT in the context of the whole psychosocial rehabilitation process, we have relied on Action Theory framework [10]. Action theory provides a comprehensive theoretical framework that describes how everyday decisions are made and has been applied to the process of prescribing medication treatments [11]. It distinguishes between (1) *action planning*, which is based on theoretical knowledge, experiential knowledge, assessment of the situation, and anticipations; (2) *decision making*, which includes the development of an intention, emotional assessment, and goal setting; (3) *operation*, which refers to the implementation of action, effect control, and feedback. Different points need to be taken into account to facilitate the choice of treatments.

**(a) Overview of Available Options**

When deciding on which treatment should be prescribed next, there must be a list of all available treatment options which can be used in the treatment of the present illness, be they first- or second-line options.

**(b) Hierarchical Sequence of Options**

The available options must then be brought into a hierarchical order with respect to the effectiveness of treatments, side effects, costs, and so on. Such hierarchies can be based on guidelines or the scientific literature.

**(c) Feasibility in the Given Context**

Next, context information must be taken into account, for example, whether an option is feasible in the specific case and treatment situation.

Reasons for nonfeasibility may include nonavailability of the treatment in a certain setting, costs, and so forth.

**(d) Utilization**

Now, information from the individual patient has been taken into account. Treatment options which have already been used in the past with the individual patient need to be clarified.

**(e) Effectiveness of Previous Treatments**

How the individual patient responded to treatments that have previously been used must be assessed.

**(f) Tolerability**

Similarly, tolerability must be assessed. Were there any side effects?

**(g) Appropriateness and/or Aggressiveness of Application**

To evaluate positive and negative effects during pretreatments, how they have been applied must be clarified. What was the maximum prescribed dose? Was it high enough for the treatment to have a chance of being effective? In summary, a judgment has to be made on the appropriateness and aggressiveness of previous treatments.

**(h) Patient Acceptance**

Important factors in treatment selection are preferences or rejections on the part of the patient. Patients like some drugs or dislike others. Whatever the reason may be, rational or irrational, this will influence patient cooperation and medication compliance.

## 4. Information for a Rational Selection

**Overview of Available Options**

**Figure 2:**
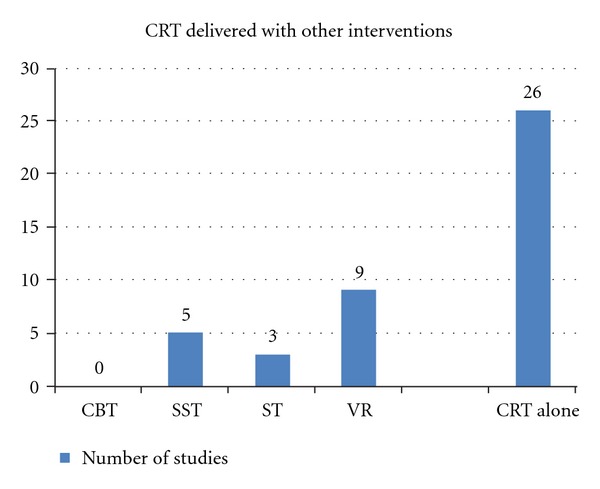
Data from the meta-analytic studies by McGurk et al. [1] and Wykes et al. [2]. CBT: Cognitive behavioral therapy; SST: social skills training; ST: skills training; VR: vocational rehabilitation; CRT alone: cognitive remediation therapy as stand-alone therapy.

To consider various psychological interventions as possible candidates for inclusion in the whole rehabilitation process, two criteria have been established. Firstly, it needs to be an evidence-based intervention with at least one published meta-analysis showing efficacy. Secondly, although it is not a necessary condition, it is valuable to take into account the studies that combine or compare CRT with other distinct psychological interventions. Surprisingly, most clinical studies included in CRT meta-analytic studies have not tested these combinations and have focused primarily on CRT as a stand-alone treatment (Figure 2). Nonetheless, at least some of these interventions can be considered as good candidates. It should be mentioned that this selection is not intended to provide a general guideline for psychosocial rehabilitation interventions and for that reason it does not account for all evidence-based interventions. It is only a proposal on how to integrate CRT with other psychosocial interventions to make CRT more effective.

**Social Skills Training (SST)**

Kurtz and Mueser [12] conducted a meta-analytic study with outcome measures from 22 studies including 1,521 clients. Results reveal a large weighted mean effect size for content-mastery exams (Cohen's *d* = 1.20), a moderate mean effect size for performance-based measures of social and daily living skills (Cohen's *d* = 0.52), moderate mean effect sizes for community functioning (Cohen's *d* = 0.52) and negative symptoms (Cohen's *d* = 0.40), and small mean effect sizes for other symptoms (Cohen's *d* = 0.15) and relapse (Cohen's *d* = 0.23). These results support the efficacy of social skills training for improving psychosocial functioning in schizophrenia. More interestingly, some aspects of the subanalysis performed by the authors led to the conclusion that there is enough evidence for the generalization of social skills training interventions from the training environment to the more complex spheres of everyday functioning.

**Social Cognition Training (SCT)**

Kurtz and Richardson [13] recently conducted a meta-analytic study to assess the efficacy of behavioral training programs designed to improve social cognitive function. A total of 19 studies consisting of 692 clients was selected from the published literature in the most important databases. With respect to social cognitive measures, weighted effect size analysis revealed some moderate-large effects of social cognitive training procedures on Facial Affect Recognition (identification, Cohen's *d* = 0.71 and discrimination, Cohen's *d* = 1.01) and small-moderate effects of training on Theory of Mind (Cohen's *d* = 0.46), while effects on social cue perception and attributional style were not significant. For measures of generalization, a weighted effect size analysis was performed and it revealed that there were moderate-large effects on total symptoms (Cohen's *d* = 0.68) and observer-rated community and institutional function (Cohen's *d* = 0.78). In summary, although the effects of social cognitive training programs on positive and negative symptoms in schizophrenia were nonsignificant, positive effects were found on different measures of affect recognition and various theories of mind components.

**Cognitive Behavioral Therapy (CBT)**

Wykes et al. [14] explored the effect sizes of current CBT trials including targeted and nontargeted symptoms, modes of action, and the effect of methodological rigor. Thirty-four trials with data in the public domain were used as source data for a meta-analysis and investigation of the effects of trial methodology using the Clinical Trial Assessment Measure. The authors found overall beneficial effects for the target symptom (33 studies; Cohen's *d* = 0.4) as well as significant effects for positive symptoms (32 studies), negative symptoms (23 studies), functioning (15 studies), mood (13 studies), and social anxiety (2 studies) with effects ranging from 0.35 to 0.44. Surprisingly, improvements in one domain were found to be correlated with improvements in other domains. Recently, a further meta-analytic study was published with special emphasis on followup. When CBT was compared with other psychological treatments at followup, there was strong evidence (with small treatment effect) that intervention has an effect on positive, negative, and general symptoms. Therapies for schizophrenia patients of at least 20 sessions had better outcomes than those that were shorter.

**Hierarchical Sequence of Options**

A number of reasons lead us to consider a hierarchy in which CRT might be the first treatment option to be considered. Firstly, cognitive impairment can act as a barrier to the other interventions [15] such as skills training [16], cognitive behavioral therapy [17], or vocational rehabilitation [18]. Neurocognitive impairments in schizophrenia have been linked to treatment response, employment status, social relationships, living status, insight into illness, therapeutic alliance, and community functioning [19, 20]. Thus, it is reasonable to suppose that cognitive impairment can also be a rate-limiting factor in some psychological treatments and that the severity of cognitive impairment may also limit its clinical benefit. Secondly, cognitive remediation therapy seems to potentiate the efficacy of other psychological interventions [21]. Finally, it has been suggested that the CRT posttraining period could be opening a critical window for aggressive adjunctive psychosocial rehabilitation [22]. On the other hand, together with work therapy, CRT plus SST is the most-tested combination and outcomes are better when they are combined [1, 2]. Consequently, social skills training (SST) and other skills training could be the second line of treatment. Finally, but just as importantly, cognitive behavioral therapy represents the next line of treatment in the flowchart. Despite convincing evidence in favor of cognitive behavior therapy (CBT) for psychosis in schizophrenia with regard to symptoms [14], its effects on functioning are not as good and for that reason some limits to its use may need to be established to optimize its implementation in clinical settings.

**Feasibility in the Given Context**

One of the most worrying deficiencies in clinical practice is the lack of access to most evidence-based psychosocial interventions. In spite of the existence of clear recommendations in the clinical guidelines for the implementation of evidence-based psychological treatments, they are certainly not sufficient. Research indicates that passive dissemination of clinical guidelines alone is generally insufficient for affecting successful implementation and improving patient outcomes [23]. Some barriers such as severe workload, time pressure, and the need for specialist staff have been described. In addition, pessimistic views of recovery for clients with psychosis have also been expressed and may affect implementation [24]. As such, providing specific flowcharts and checklists based on the guidelines that allow collaborative decision making between patients and clinicians can be of some help. Finally, although some promising data on economic considerations associated with CRT have already been published [25], studies analysing cost-effectiveness are conspicuous by their absence.

**Utilization and Effectiveness of Previous Treatments**

It seems absolutely necessary to establish a complete history of previous treatments and how effective they had been. Moreover, a comprehensive history of education, social activities, and work history can be of help in choosing the right interventions to establish the hypothetical relationship between cognitive deficits and functioning when taking all the contextual variables into account. Experience in previous rehabilitation programs can provide relevant information which allows personalization of the intervention program. Some decisions about the use of paper-and-pencil or computer tasks, group or individual format, or even the characteristics of the cognitive exercises can be made using this personal information. Furthermore, their social and work history might help to define daily functioning goals in a more personalized way.

**Tolerability and Appropriateness or Aggressiveness of Application**

Unfortunately, no study has specifically tested the question of the optimal dose of treatment in terms of number of sessions or hours of treatment. Nonetheless, some data on the question of treatment intensity have been published by the Alice Medalia group [26]. In a study where patients followed cognitive training, they compared the change in standard scores between those patients taking less than 128 days and those taking more than 128 days to complete the training, the median being 128 days/4.5 months for the whole sample. It was found that patients taking longer than the median time to complete training benefited significantly less than those completing the training in a shorter period of time. The effect size for patients undergoing high-intensity treatment (completing training in less than 128 days) was quite large (Cohen's *d* = 1.46), whereas the effect size for the group of patients receiving lower-intensity treatment (more than 128 days) was small (*d* = 0.26). What this remarkable difference in effect sizes underscores is the important role that treatment intensity has in the gains patients make in cognitive remediation. Thus, it is important to stress that CRT should be delivered intensively at least two days a week over four months.

**Patient Acceptance**

High rates of early dropout from psychosocial rehabilitation programs have been described [27]. For cognitive and social rehabilitation programs different causes of attrition have been proposed: age at start of treatment, number of hospitalizations, and verbal fluency were linked to the ability of clients with schizophrenia to participate in community-based, intensive cognitive and social rehabilitation programs, even when other demographic, neurocognitive and symptom variables were accounted for. Race and ethnicity, sex, education, parental education, age at onset, symptoms, and cognitive factors of sustained attention, verbal memory, and problem solving were not related to attrition status in the current study. Medalia and Saperstein [28] suggested that voluntary attendance is a measure of intrinsic motivation for treatment and this indicates that motivation is an important patient characteristic when aiming for a positive treatment outcome. Thus, case formulation and intervention based on Action Theory can be of some help in enhancing motivation. However, when motivation is so low that it prevents participation in the rehabilitation process, it will be necessary to previously target it directly.

## 5. The Neurocognitive Behavioral Approach

Taking into account the aforementioned discussed data on the variety of treatments and the whole psychosocial rehabilitation process, an evidence-based, person-centered approach for delivering cognitive remediation with other psychological treatments is presented here. There are many treatment guidelines which summarize general scientific evidence on how to treat a particular illness. However, they can not take into account individual patients and their particular treatment history. This guideline is based on the principals of the neurocognitive behavioral approach established elsewhere by Penadés and Gastó [29].

- It is an empirical approach that incorporates any sort of methodologies, learning techniques, rehabilitation programs, software, or paper-and-pencil tasks provided that their efficacy has previously been demonstrated in controlled studies.
- Rehabilitation treatment should focus on improving neurocognition but the main target is to ameliorate associated psychosocial disability.
- Rehabilitation treatment must be customized for each patient and should focus on those targets considered to be important by the patient.
- Rehabilitation targets should be agreed with the patient and should be based on their capabilities, needs, and current social environment
- This approach is called “neurocognitive behavioral” since it proposes comprehensive treatment of neurocognitive aspects but does not overlook emotional, functional, and psychological ones.

The use of flowcharts based on these principles can also support patient cooperation in the whole rehabilitation process. It allows clinician and patient to make a rational decision together on what to do next, which is the core idea of shared decision making. Thus, to integrate CRT into the whole psychosocial rehabilitation context, the following steps might be considered.

**Figure 3:**
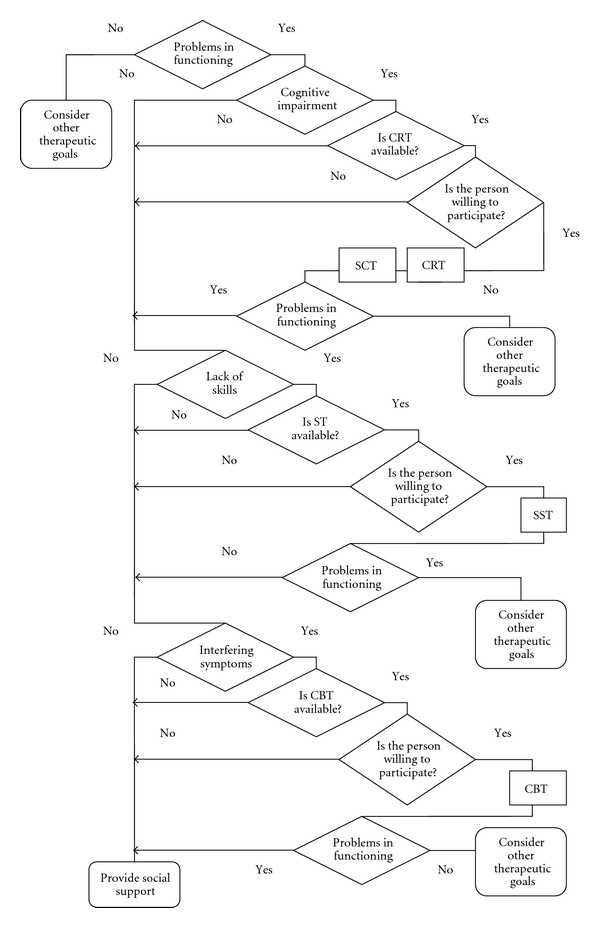
Flowchart to integrate cognitive remediation with other psychological interventions. CRT: cognitive remediation therapy; SCT: social cognition therapy; SST: social skills training; CBT: cognitive behavioral therapy.

1. Comprehensive initial assessment: along with neurocognitive functioning, other aspects such as history of education, work history, social skills competence, presence of interfering symptoms, expectations of self-efficacy, and motivation level need to be assessed. Additionally, other aspects influencing daily functioning need to be taken into account in the form of a functional analysis. Therefore, characteristics regarding current social and familial network, availability of community resources, and, above all, patients' personal preferences also need to be considered.
2. Identification of personal goals: patient and therapist must define relevant goals in a collaborative framework not only in terms of neurocognition but also in terms of daily functioning. By paying special attention to the relationship between cognitive deficits and subsequent problems in functioning, the therapist will be able to help the patient to create a problem list. It is essential that patients consider the goals of the intervention as being truly relevant to their daily lives.
3. Case formulation and tailoring of the intervention: formulation is a way of generating a hypothesis that could be tested through the application of treatment interventions. Thus, after the identification of personal goals, an intervention plan can be formulated taking into account both the initial assessment and the interventions flowchart (Figure 3). Both action theory framework (action planning, decision making, and operation) and the characteristics of the various psychosocial interventions (efficacy, intensity, methods, suitability, etc.) need to be discussed with the patient to set up the tailored intervention plan.

## 6. Specific Cognitive Abilities That Should Be Targeted to Improve Functioning

As has been suggested before, improved cognitive function can lead to improved daily functioning in the context of psychological interventions. However, the identification of the cognitive domains that have to be targeted to improve functioning is still incomplete. As mentioned above, the impact of CRT on functioning is important because the primary rationale for this therapy is to improve not only cognition but also psychosocial functioning [30]. Surprisingly, most CRT clinical studies have not tested this hypothesis until recently and have focused primarily on cognitive performance [1, 2]. Obviously, an understanding of the links between cognitive change and functional improvement can be crucial in identifying appropriate cognitive targets for treatment leading to functional improvement.

In two studies, Reeder et al. [31] published some surprising results. Cognitive functions which usually show significant cross-sectional associations with social functioning are not the same as those associated with improvement in functioning in the context of CRT. In the first study, it was found that while the “response inhibition speed” factor was associated with social functioning at baseline, change in a different factor predicted social functioning change following cognitive remediation therapy (CRT). In the second study [32], a relationship at baseline was found between social functioning and various cognitive domains such as verbal working memory, response inhibition, verbal long-term memory, and visual spatial long-term memory, but not schema generation. Surprisingly, it was the improvement in schema generation which predicted improved social functioning. From the two studies, it can be concluded that cross-sectional associations between cognitive functions and social functioning may not be an appropriate approach for selection of cognitive targets for intervention. Even though selecting the CRT cognitive targets on the basis of cognitive skills that appear to predict functional outcome in schizophrenia sounds logical, it could be misleading. As such, while it has been generally assumed that improved cognition will lead to improved functional outcome, the nature of this putative link is far from clear. 

Penadés et al. [33] conducted research to investigate the neurocognitive changes occurring in the context of CRT and tried to identify which of those changes leads to improvements in daily functioning. This study used data collected as part of a randomized, controlled trial investigating a CRT program in a partner study [34]. The trial recruited 52 schizophrenia patients between the ages of 27 and 42 who had been in touch with psychiatric services for at least 10 years; composing sample with predominant negative symptoms and cognitive impairments. Of these participants, 40 were randomized to receive either CRT or a control psychological treatment (CBT) where neurocognition was not targeted. At baseline, daily functioning was significantly associated with verbal memory. Surprisingly, improvement in executive function, but not in verbal memory, predicted improved daily functioning among people with chronic schizophrenia who had current negative symptoms and evidenced neuropsychological impairments. Notwithstanding, the statistical mediation model found that social improvement caused by executive changes is expressed indirectly through improvement in verbal memory (*F*_(2/31)_ = 33.308, *P* < 0.001). Thus, the direct model, as the name suggests, represented the prediction of social improvement from the change in executive function directly. None of the executive measures, such as change in psychomotor speed, change in nonverbal memory, or change in working memory add significant explanatory power to the effect of executive change in the social improvement function equation. These results confirm that there is no evidence for a simple direct relationship between cognition and separate aspects of social functioning. Consequently, even if people have impairments in multiple cognitive domains, executive functioning still needs to be the target of the intervention.

## 7. Conclusion

It has been established that with CRT neurocognitive impairments can be ameliorated and some improvement in social functioning can also be expected. To achieve these results it is crucial that CRT is based on the teaching of cognitive strategies and that it involves some cognitive practice. CRT needs to be carried out in the context of broader psychosocial rehabilitation involving the learning of other communication, social, and self-control skills. Unfortunately, little is known about how to integrate the different rehabilitation tools in a broader rehabilitation program. Based on the neurocognitive behavioral approach and action theory framework, and obviously on published meta-analytic studies, a hierarchical flowchart has been provided to integrate CRT with other evidence-based psychological therapies. Finally, it is important to take into account that to improve functioning with CRT together with other impaired cognitive functions, executive function also needs to be specifically targeted.
